# Supplementary material for: Inhibitory Effect of Polyphenols from the Whole Green Jackfruit Flour against α-Glucosidase, α-Amylase, Aldose Reductase and Glycation at Multiple Stages and Their Interaction: Inhibition Kinetics and Molecular Simulations
Source: Molecules. 2022 Mar 14;27(6):1888. doi: 10.3390/molecules27061888 (PMC8949615; doi:10.3390/molecules27061888)
Supplement: Supplementary file 1 [file molecules-27-01888-s001.zip › molecules-1597673-supplementary.pdf]

Supplementary Materials

Inhibitory Effect of Polyphenols from the Whole Green Jackfruit Flour against α-Glucosidase, α-Amylase, Aldose Reductase and Glycation at Multiple Stages and Their Interaction: Inhibition Kinetics and Molecular Simulations

Tejaswini Maradesha [1], Shashank M. Patil [1], Khalid Awadh Al-Mutairi [2], Ramith Ramu [1,*], SubbaRao V. Madhunapantula [3] and Taha Alqadi [4]

1 Department of Biotechnology and Bioinformatics, School of Life Sciences, JSS Academy of Higher Education and Research, Mysuru 570015, Karnataka, India; tejaswini@jssuni.edu.in (T.M.); shashankmpatil@jssuni.edu.in (S.M.P.)
2. Biology Department, Faculty of Science, University of Tabuk, Tabuk 71491, Saudi Arabia; kmutairi@ut.edu.sa
3. Center of Excellence in Molecular Biology and Regenerative Medicine (CEMR; A DST-FIST Supported Center), Department of Biochemistry (A DST-FIST Supported Department), JSS Medical College, JSS Academy of Higher Education and Research, Mysore 570015, Karnataka, India; mvsstsubbarao@jssuni.edu.in
4. Department of Biology, Adham University College, Umm Al-Qura University, Makkah 21955, Saudi Arabia; taqadi@uqu.edu.sa
* Correspondence: ramith.gowda@gmail.com; Tel.: +91-9986-380-920, Fax: +91-821-2548394

Table S1. Inhibitory potential and Antioxidant activity of Crude extracts and its respective fractions of *green jackfruit flour* against α-amylase, α-glucosidase and aldose reductase enzymes.

Extracts (Ext.)/Fractions (Fr.)	Enzyme inhibition IC_{50} ^{x} (μg/ml)			Antioxidant activity EC_{50} ^{x} (μg/ml)		
	α-amylase	α-glucosidase	aldose reductase	DPPH	ABTS	Superoxide
**Chloroform (Ext.)**	43.25±1.13	24.50±0.25	8.95±0.22	35.50±0.88	35.00±0.99	66.00±2.79
**Ethyl Acetate (Ext.)**	37.00±0.59	15.25±1.05	9.75±0.47	36.88±1.00	32.02±0.55	50.55±0.35
**Acetone (Ext.)**	31.25±1.11	15.00±0.00	7.86±0.55	30.55±0.54	27.85±1.11	52.78±0.06
**Methanol (Ext.)**	**28.00±0.03**	**10.00±0.14**	**3.75±0.75**	**24.30±0.82**	**20.80±1.32**	**44.50±2.40**
**Water (Ext.)**	46.50±1.25	31.00±0.26	9.99±0.99	45.00±0.75	37.45±0.37	58.00±0.55
**Chlorofrom (Fr.)**	39.25±2.20	15.06±0.22	6.40±0.42	30.56±0.25	28.88±0.90	50.00±0.77
**Ethyl Acetate (Fr.)**	**27.80±0.06**	**09.55±0.87**	**3.60±0.00**	**24.02±1.87**	**20.01±0.33**	**44.06±1.78**
**n-butanol (Fr.)**	34.50±0.89	16.25±0.28	5.55±0.29	32.25±1.05	28.85±0.42	48.88±0.65
**Methanol (Fr.)**	33.00±1.02	14.00±1.07	5.00±0.58	30.55±0.54	25.55±0.22	47.00±0.00
**Cf:Me (35-43)**	30.00±0.56	11.05±2.00	4.75±0.88	26.25±0.44	21.25±0.35	33.33±0.33
**Cf:Me (44-52)**	29.50±0.78	11.00±0.66	4.65±0.22	25.00±0.22	24.44±0.42	35.00±0.99
**Cf:Me (53-61)**	29.00±0.55	10.75±0.75	4.00±0.54	22.05±1.02	19.50±0.10	32.00±2.02
**Cf:Me (62-70)**	28.50±1.00	10.00±1.00	4.20±0.47	20.00±0.60	15.00±0.40	33.00±3.00
**Cf:Me (71-79)**	**26.90±0.05**	**8.00±0.40**	**3.10±0.33**	**18.50±0.08**	**12.44±1.60**	**30.13±2.05**
**Cf:Me (80-88)**	27.85±0.25	11.00±0.55	5.00±0.10	21.00±0.55	14.00±0.04	31.11±1.11
**Cf:Me (89-96)**	28.00±0.54	10.00±0.25	4.00±0.15	19.50±0.24	13.00±0.00	31.25±0.77
**Cf:Me (97-101)**	**25.25±1.00**	**7.50±1.05**	**3.00±0.00**	**16.00±0.13**	**11.40±2.04**	**28.00±1.19**

^{x} *Values are reported as mean ± SE.*

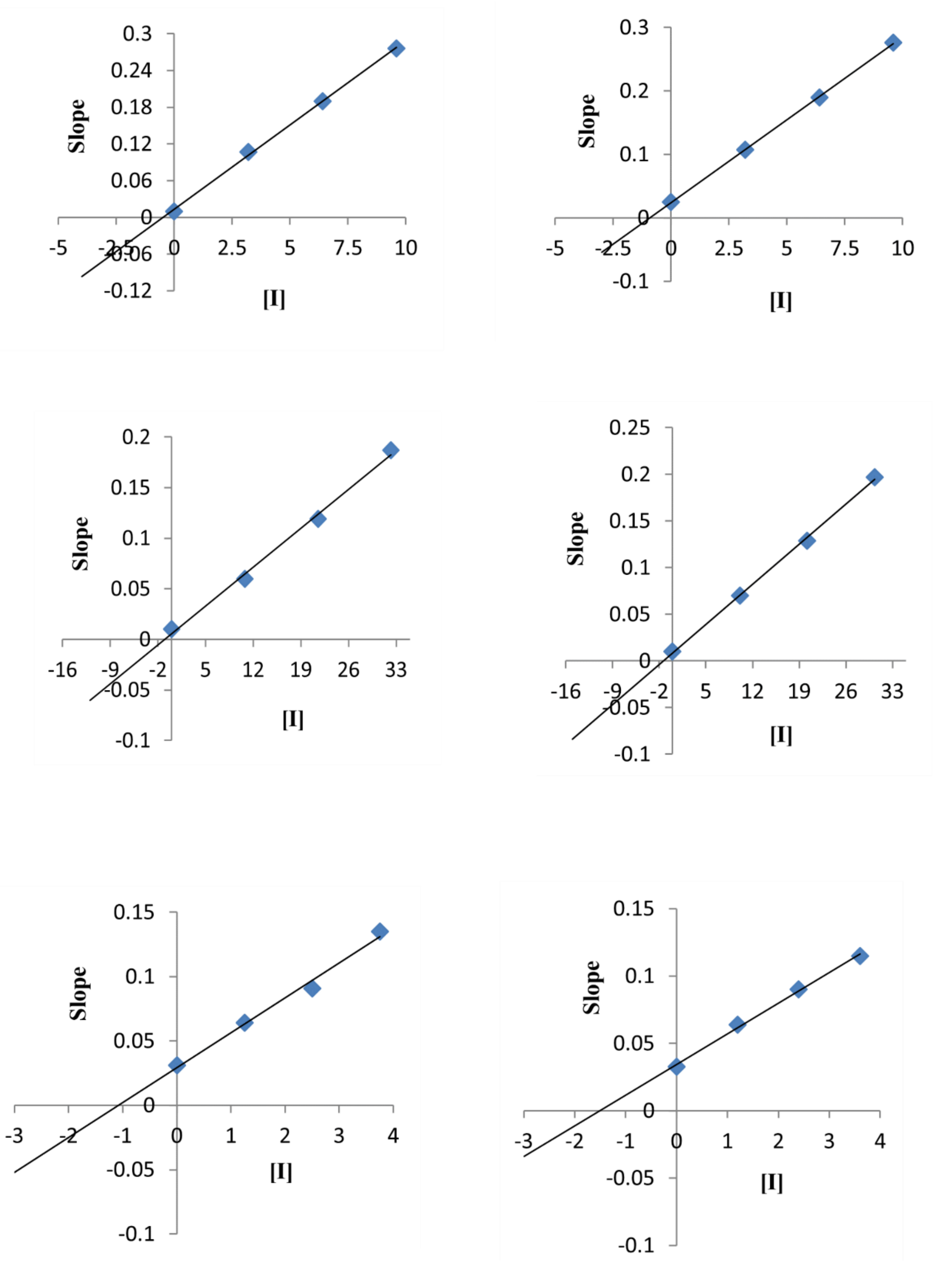

Figure S1. Determination of Ki from secondary plots of double reciprocal plots against α-glucosidase (A & B), α-amylase (C & D) and aldose reductase (E & F) inhibition by caffeic acid and syringic acid

Molecular dynamics simulations

Molecular dynamics simulation for the protein backbone atoms as well as protein-ligand complexes has been performed in triplicates. The same pattern of the results was obtained in the 3 trials conducted. The average results of the MD trajectories were put into the main article. The triplicates with the similar amount and patterns of data indicate that the simulation run is without flaws and are stable in nature.

Figure S2 and **S3** depict the results obtained for the simulation of α-glucosidase. Similarly, **Figure S4** and **S5** represent the MD simulation results of α-amylase, and **Figure S6** and **S7** represent the same for HAR. After performing the molecular dynamics simulations, expected results were taken for consideration.

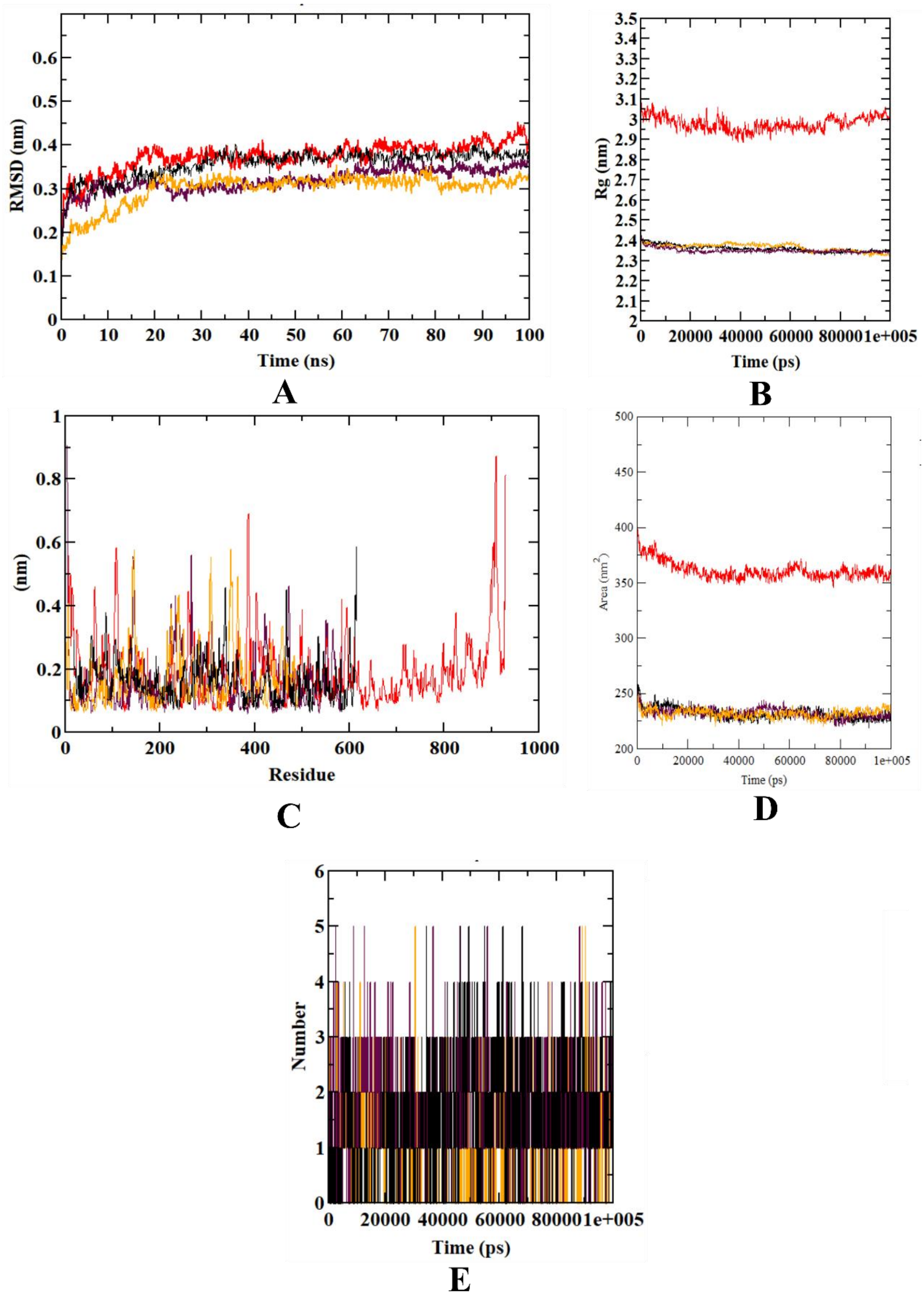

Figure S2. Visualization of dynamics simulation of experimental compounds with α-glucosidase. A) protein-ligand complex RMSD, B) Rg, C) RMSF, D) SASA, E) ligand hydrogen bonds. Red: protein backbone atoms, maroon: protein-caffeic acid complex, black: protein-syringic acid complex, orange: protein-acarbose complex.

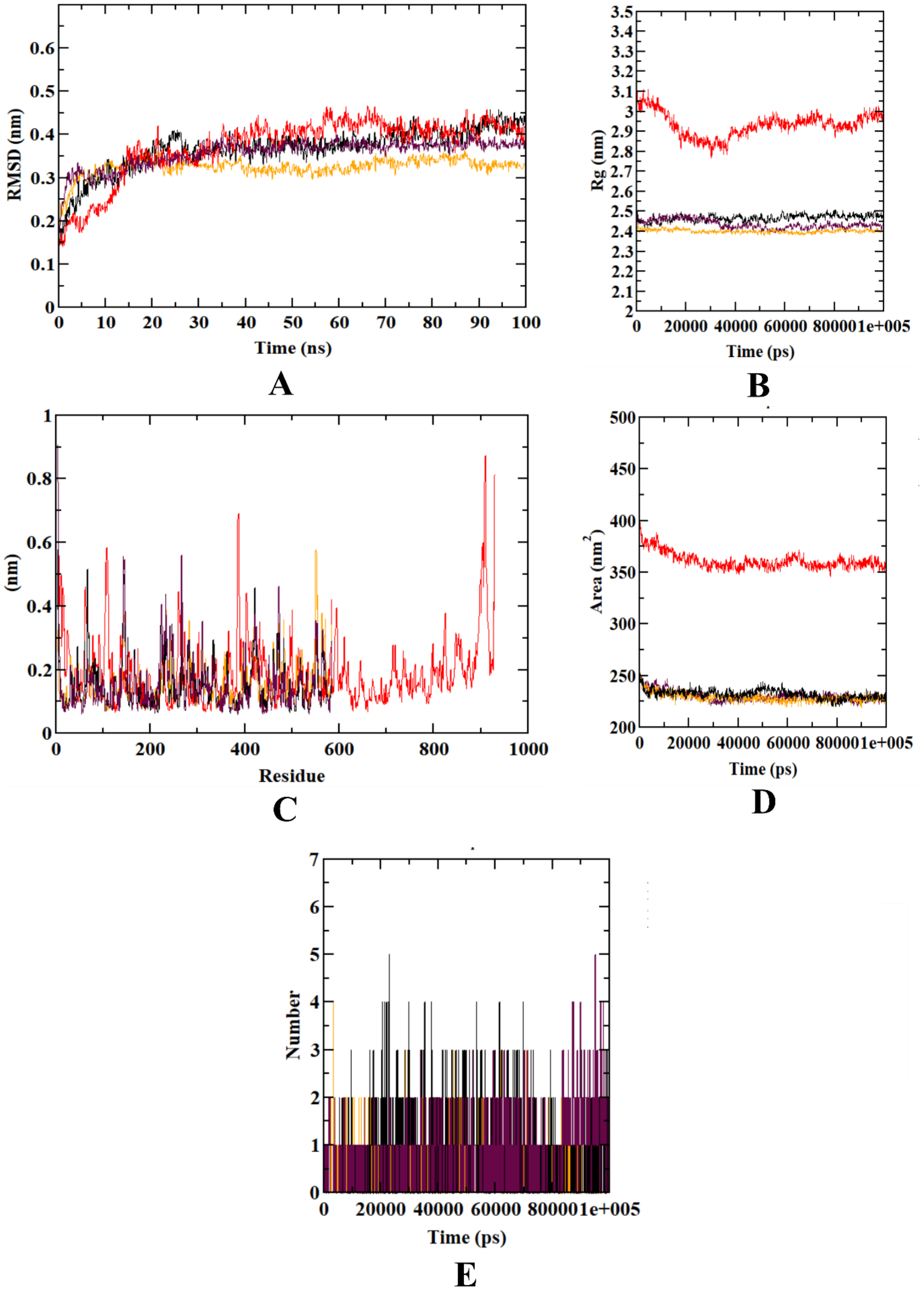

Figure S3. Visualization of dynamics simulation of experimental compounds with α-glucosidase. A) protein-ligand complex RMSD, B) Rg, C) RMSF, D) SASA, E) ligand hydrogen bonds. Red: protein backbone atoms, maroon: protein-caffeic acid complex, black: protein-syringic acid complex, orange: protein-acarbose complex.

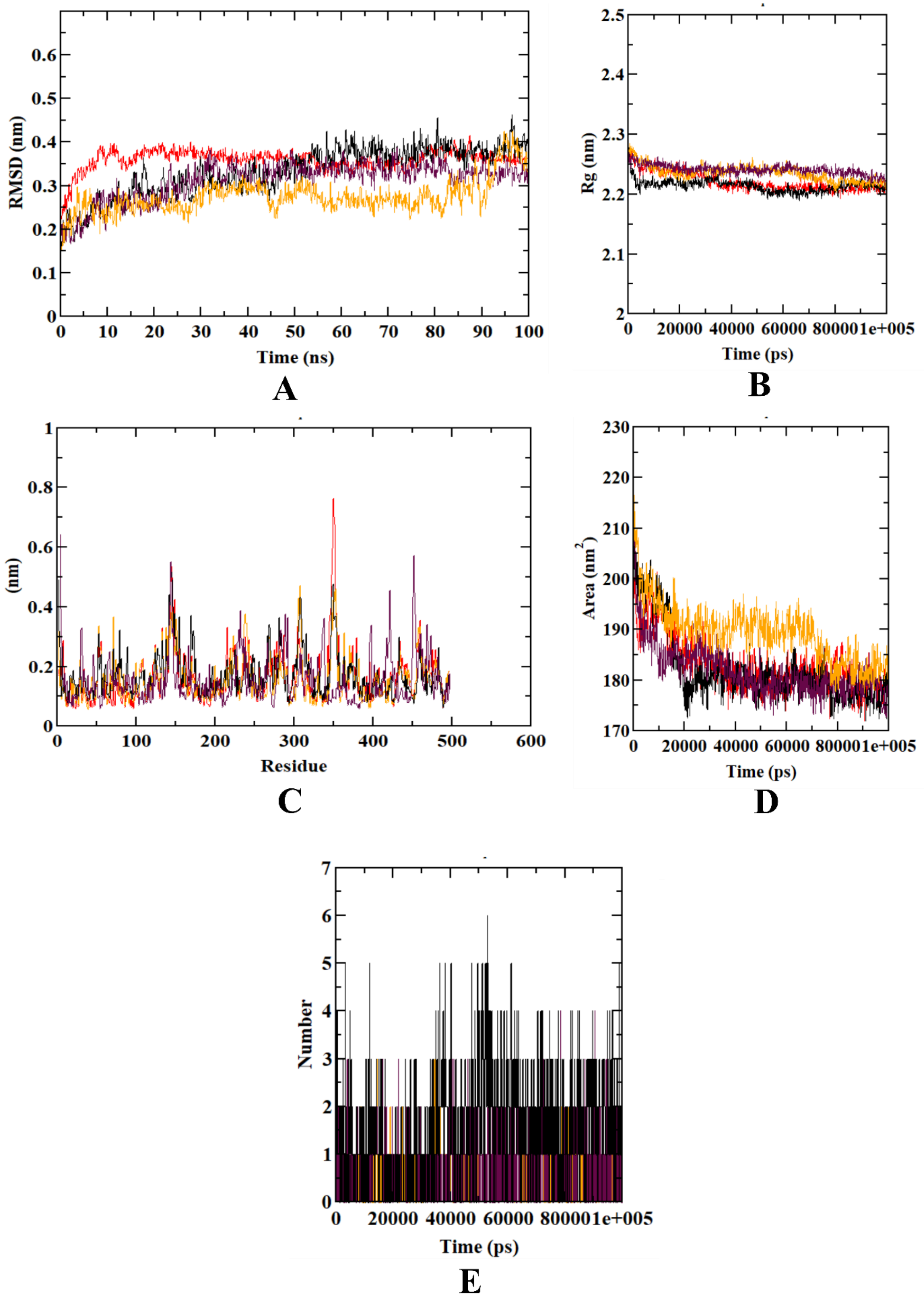

Figure S4. Visualization of dynamics simulation of experimental compounds with α-amylase. A) protein-ligand complex RMSD, B) Rg, C) RMSF, D) SASA, E) ligand hydrogen bonds. Red: protein backbone atoms, maroon: protein-caffeic acid complex, black: protein-syringic acid complex, orange: protein-acarbose complex.

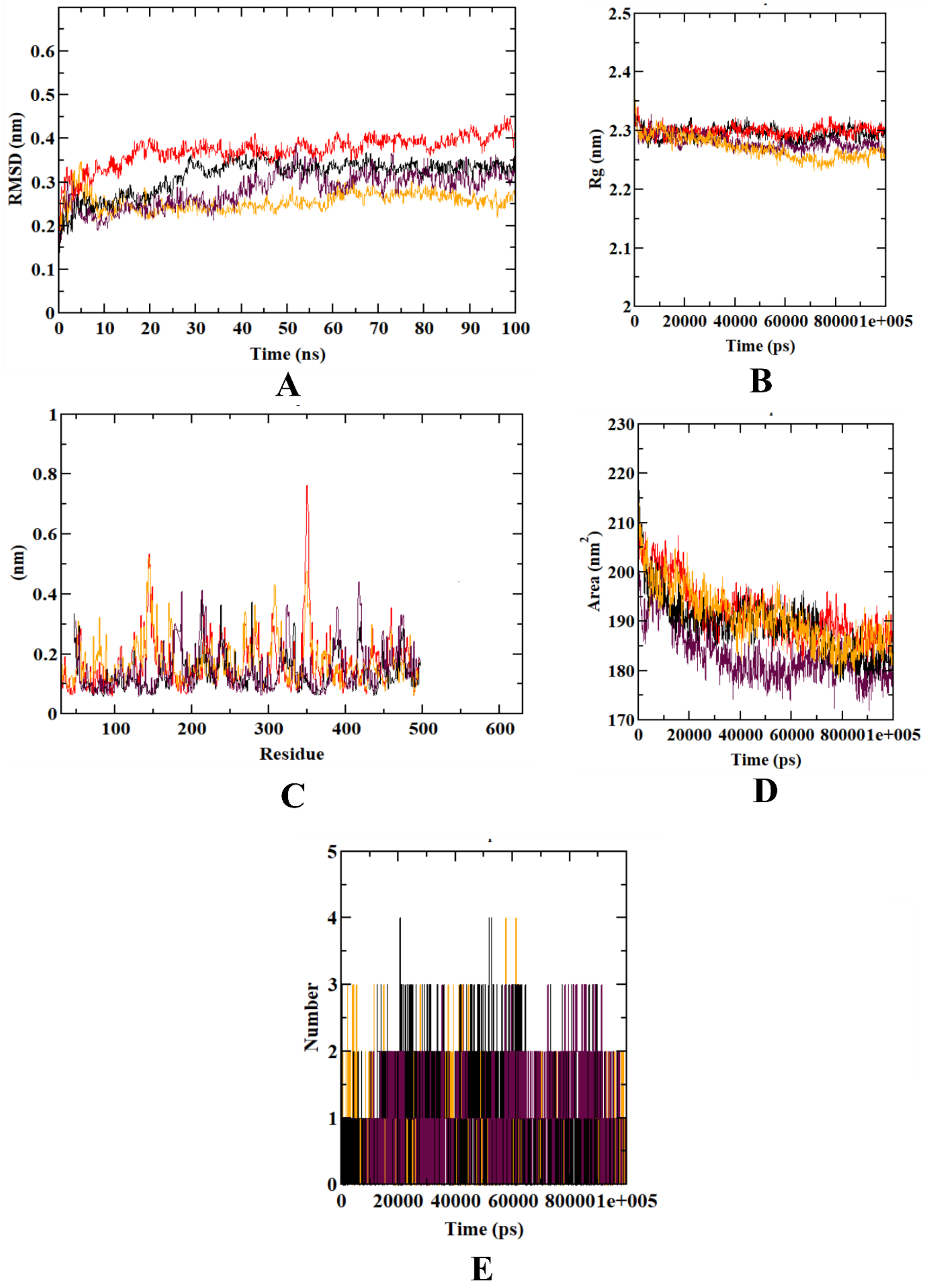

Figure S5. Visualization of dynamics simulation of experimental compounds with α-amylase. A) protein-ligand complex RMSD, B) Rg, C) RMSF, D) SASA, E) ligand hydrogen bonds. Red: protein backbone atoms, maroon: protein-caffeic acid complex, black: protein-syringic acid complex, orange: protein-acarbose complex.

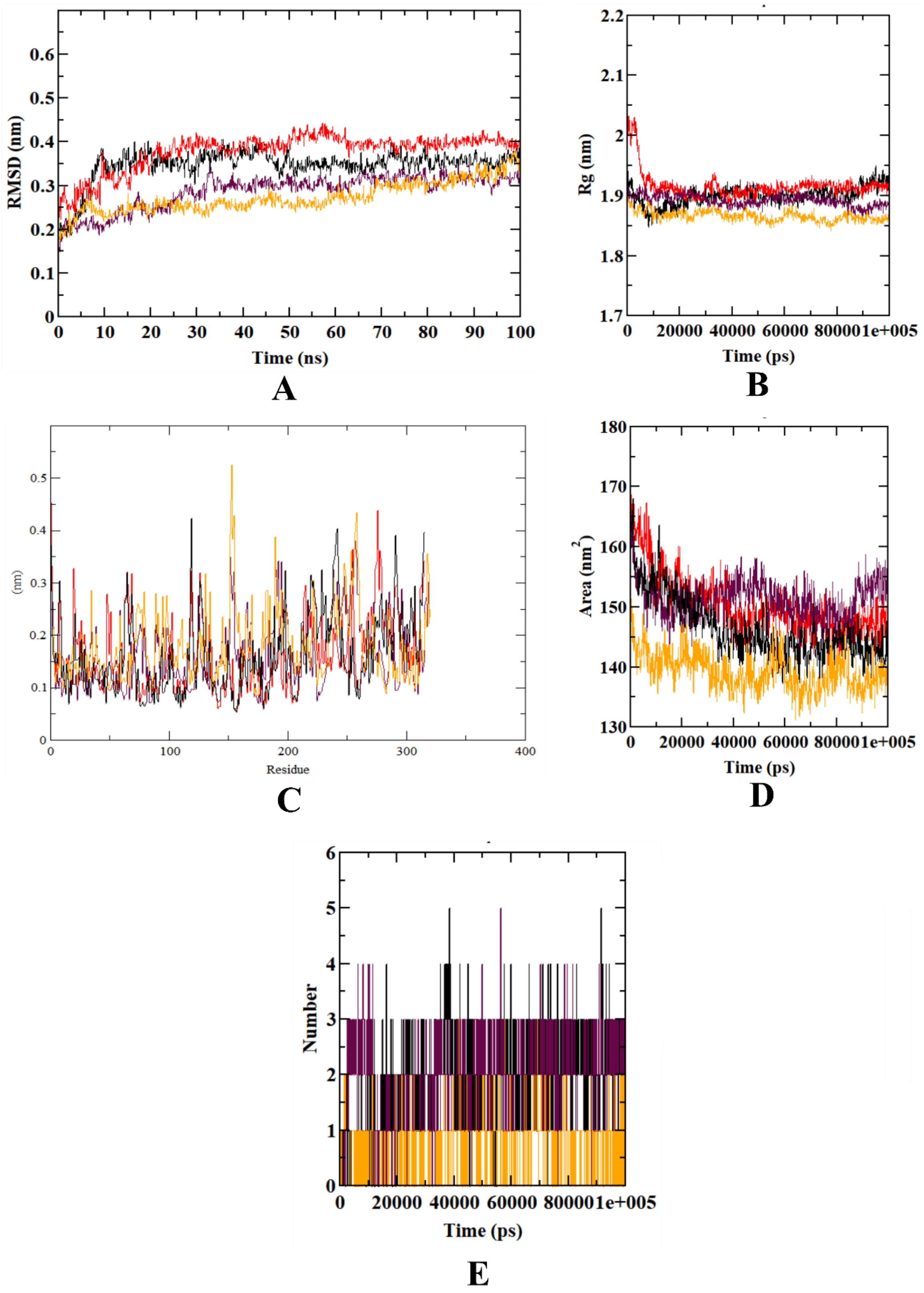

Figure S6. Visualization of dynamics simulation of experimental compounds with HAR. A) protein-ligand complex RMSD, B) Rg, C) RMSF, D) SASA, E) ligand hydrogen bonds. Red: protein backbone atoms, maroon protein-caffeic acid complex, black: protein-syringic acid complex, orange: protein-quercetin complex.

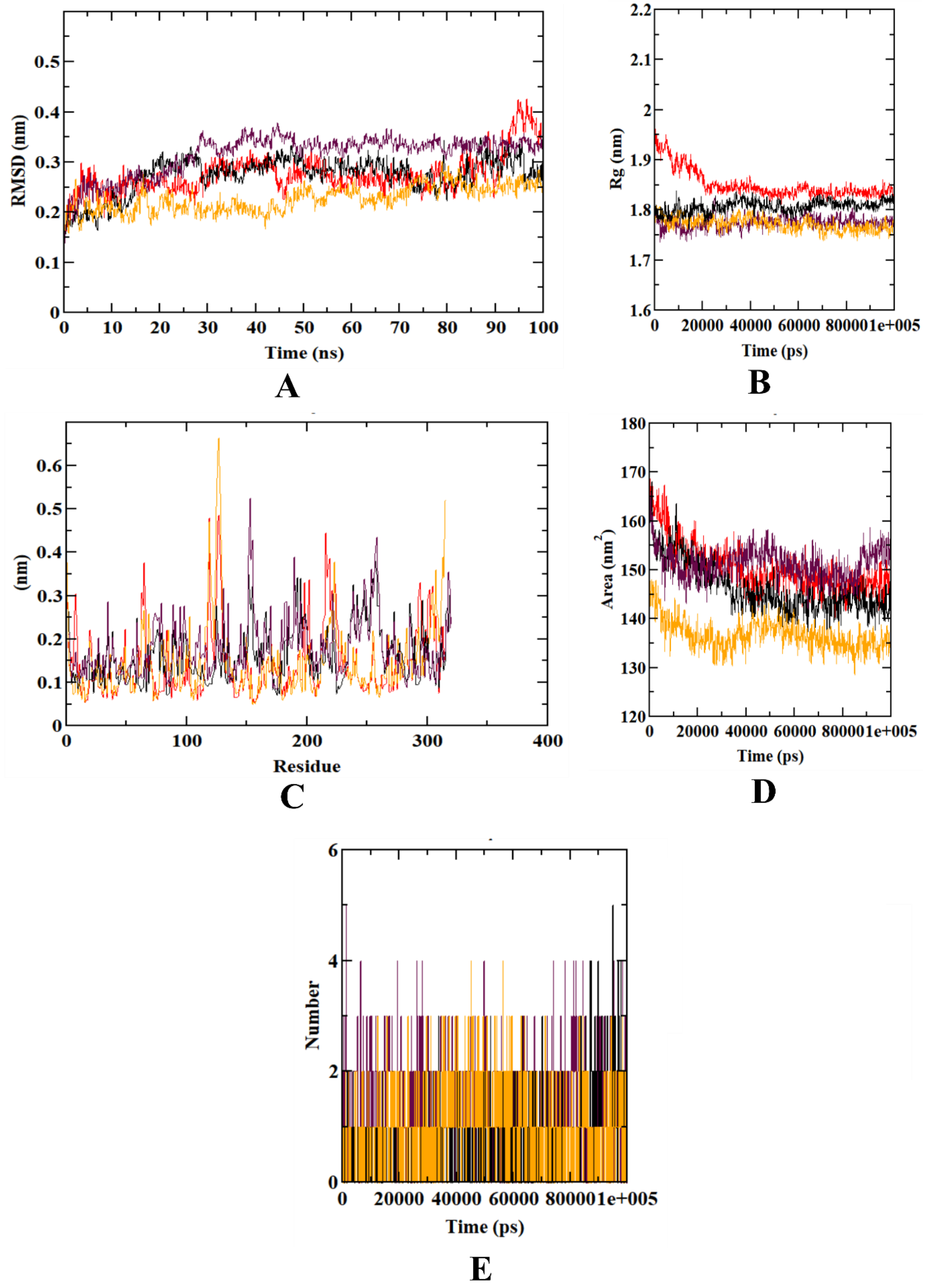

Figure S7. Visualization of dynamics simulation of experimental compounds with HAR. A) protein-ligand complex RMSD, B) Rg, C) RMSF, D) SASA, E) ligand hydrogen bonds. Red: protein backbone atoms, maroon protein-caffeic acid complex, black: protein-syringic acid complex, orange: protein-quercetin complex.

Hydrogen bond residue mapping for the catalytic mechanism assessment

To evaluate the catalytic mechanism of the interactions formed, hydrogen bond residue mapping of the residues was performed using the 'md distance' utility available in GROMACS 2018.1 software package. Through this approach, distance between the amino acid residue atoms and ligand atoms was calculated for the time period of 50 ns (last 50 ns of the 100 ns simulation run time). Since hydrogen bonds were included as key parameter in virtual screening of the ligands, residues bound only with hydrogen bonds were considered for the mapping. The cut-off value for hydrogen bond was set for 3.5 Å, according to a study by **Morris et al. (2014) [1]**. The bonds with bond length more than 3.5 Å were not considered as feasible. However, our molecular dynamics studies fit into this this criterion. The figures indicate the individual plots put for each binding residue, that was bound with hydrogen bond. **Figure S8** depict the hydrogen mapping of all the α-glucosidase residues bound to the experimental molecules with hydrogen bonds. Whereas, **Figure S9** and **Figure S10** indicate the same for α-amylase and human aldose reductase, respectively. In case of human aldose reductase **(Figure S10A; black coloured plot)**, hydrogen atom of caffeic acid bound with seaborgium (SG) atom of TYR 309 was not able to fit into the cut-off value, therefore the interaction is considered to be unstable.

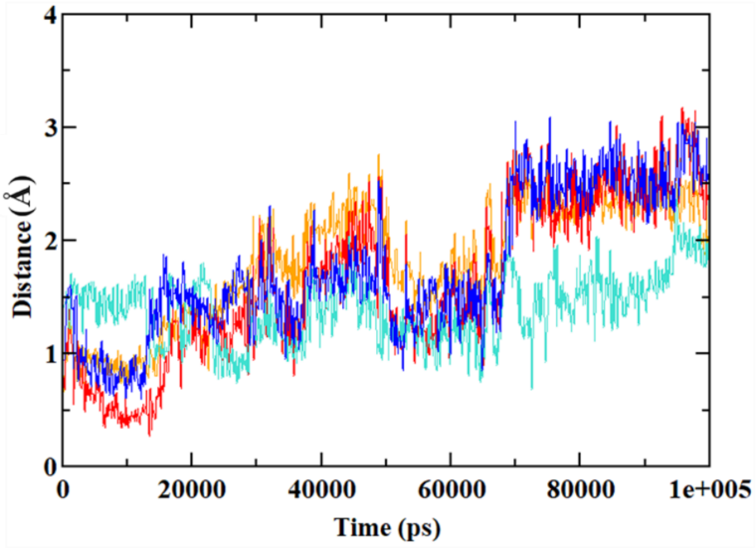

A

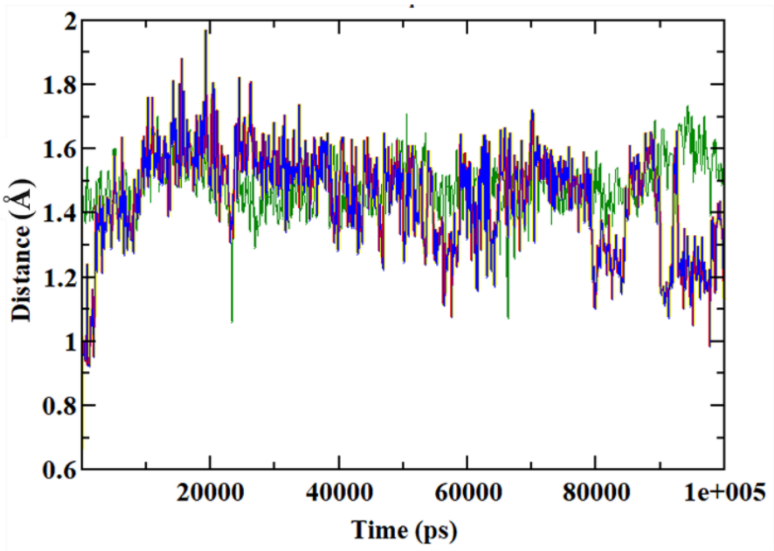

B

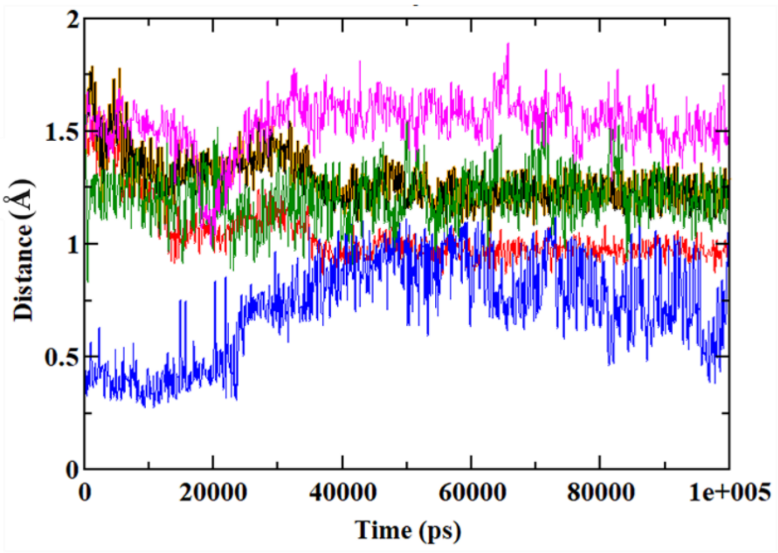

C

Figure S8. Hydrogen bond mapping of all the α-glucosidase binding residues bound to A) caffeic acid, B) syringic acid, and C) acarbose through hydrogen bond.

Table S2. (figure legend for Figure S8).

Figure	Ligand	Binding residue	Residue atom	Ligand atom	Plot colour
**A**	Caffeic acid	ASP 214	O	H	Orange
		GLU 276	O	H	Red
		ARG 312	H	O	Turquoise
		ASP 349	O	H	Blue
**B**	Syringic acid	ASP 68	H	C	Green
		PHE157	O	H	Yellow
		GLU 276	O	H	Red
		ASP 439	H	O	Blue
**C**	Acarbose	HIS 239	C	H	Red
		ASN 241	H	O	Blue
		PRO 309	H	C	Black
		PRO 309	O	H	Yellow
		ASP 408	O	H	Green
		ARG 439	H	O	Magenta

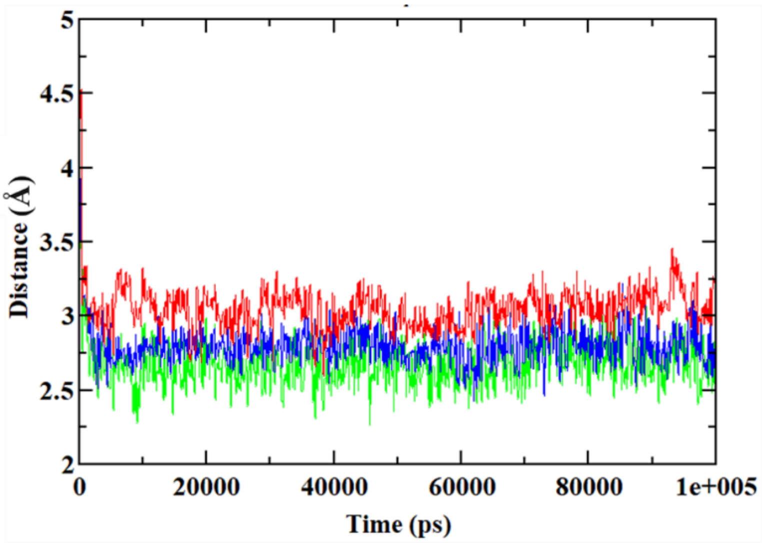

A

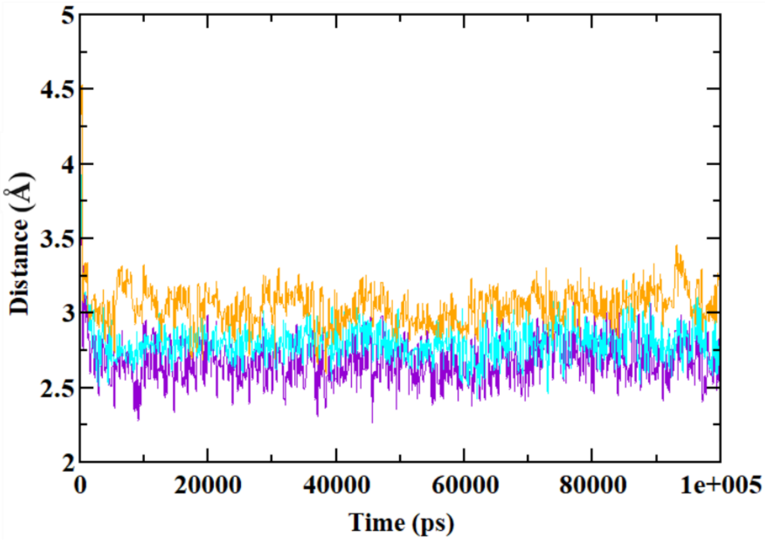

B

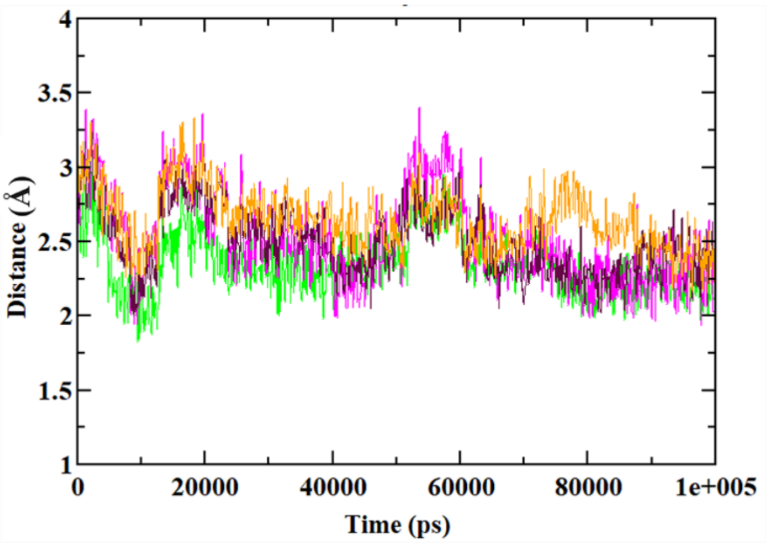

C

Figure S9. Hydrogen bond mapping of all the α-amylase binding residues bound to A) caffeic acid, B) syringic acid, and C) acarbose through hydrogen bond.

Table S3. (figure legend for Figure S9).

Figure	Ligand	Binding residue	Residue atom	Ligand atom	Plot colour
**A**	Caffeic acid	TYR 62	C	H	Green
		GLN 63	H	O	Blue
		ASP 197	O	H	Red
**B**	Syringic acid	TYR 62	H	O	Violet
		GLU 233	H	O	Blue
		ASP 300	C	H	Orange
**C**	Acarbose	ASP 197	O	H	Green
		GLU 233	O	H	Magenta
		GLU 233	H	O	Maroon
		ASP 300	H	C	Orange

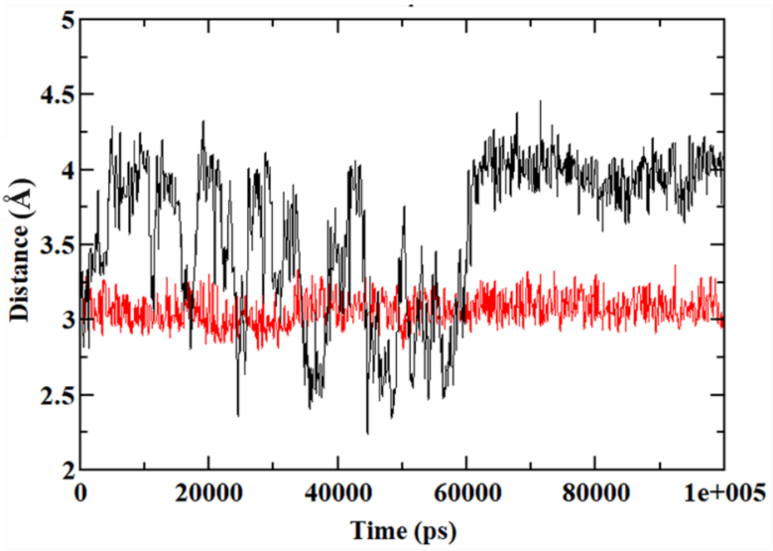

A

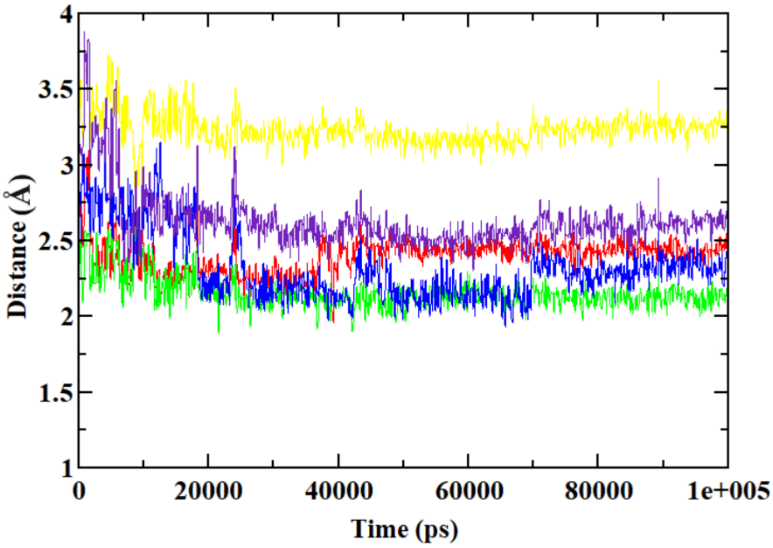

B

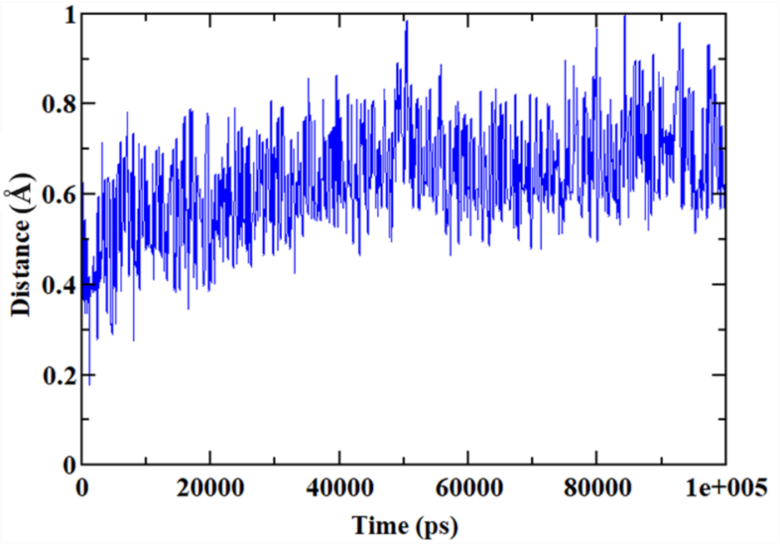

C

Figure S10. Hydrogen bond mapping of all the human aldose reductase binding residues bound to A) caffeic acid, B) syringic acid, and C) acarbose through hydrogen bond.

Table S4. (figure legend for Figure S10.)

Figure	Ligand	Binding residue	Residue atom	Ligand atom	Plot colour
**A**	Caffeic acid	CYS 80	SG	H	Red
		TYR 309	H	O	Black
**B**	Syringic acid	TRP 20	H	O	Red
		TYR 48	H	O	Green
		HIS 110	H	O	Blue
		HIS 110	C	O	Yellow
		CYS 298	H	C	Indigo
**C**	Quercetin	CYS 298	O	H	Blue

Carcinogenicity of quercetin

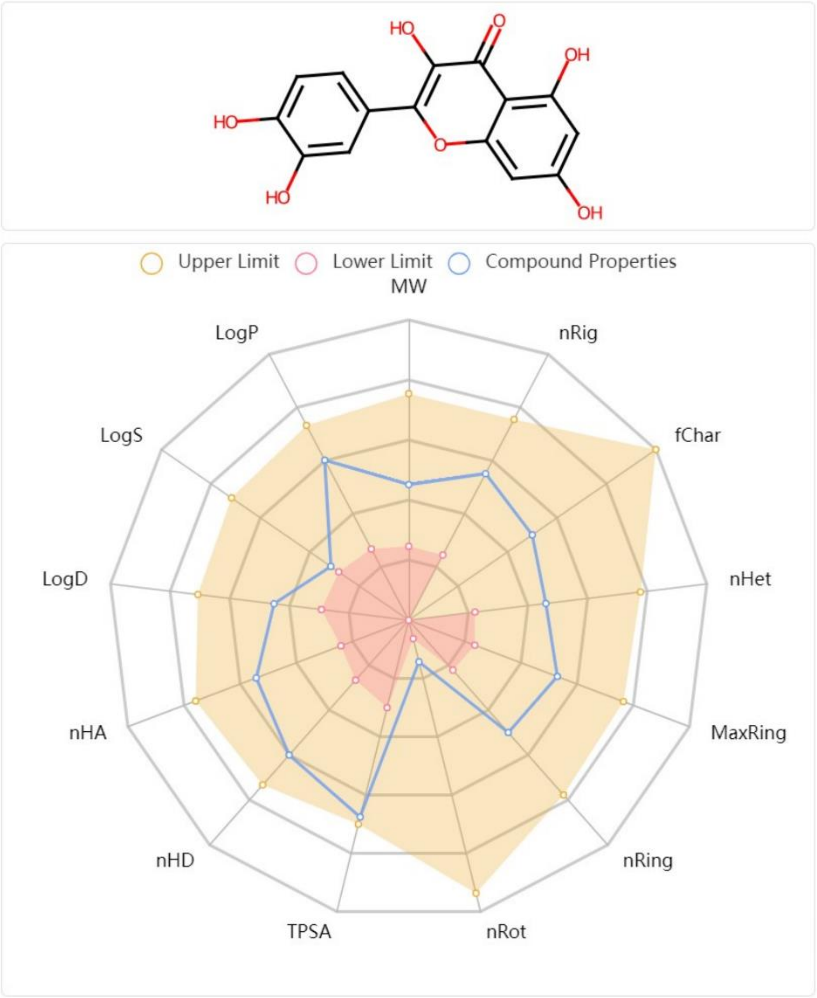

A

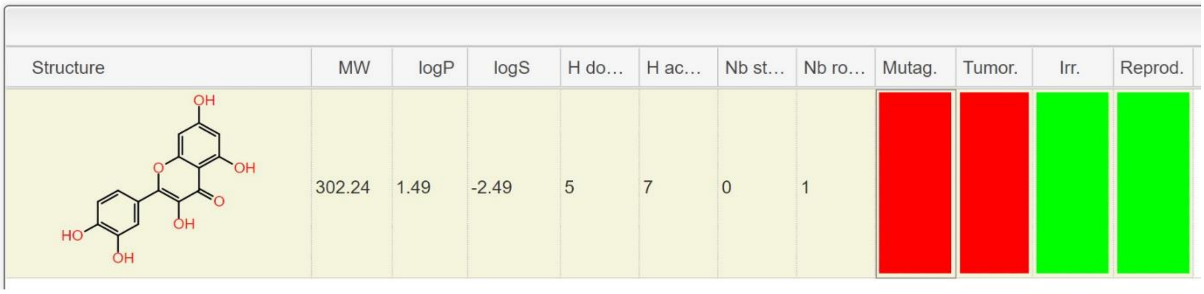

Structure	MW	logP	logS	H do…	H ac…	Nb st…	Nb ro…	Mutag.	Tumor.	Irr.	Reprod.
	302.24	1.49	-2.49	5	7	0	1				

B

Figure S11. A) Pharmacokinetic mapping of quercetin obtained from ADMETlab 2.0, B) pharmacokinetic properties showing mutagenicity and tumerigenicity, obtained from OSIRIS Property Explorer.

Authors performed an *in silico* pharmacokinetic analysis of quercetin again to provide the suitable information about its carcinogenic property. Apart from ADMETlab 2.0, which was used in the initial analysis, OSIRIS Property Explorer web tools was also used this time. During the analysis, quercetin was found to be in the upper limit of the overall pharmacokinetic cut-off values **(Figure S11A)**. However, results from OSIRIS Property Explorer **(Figure S3)** showed that the molecule is mutagenic and tumorigenic. The numerical data obtained from ADMETlab 2.0 **(Table 7)** supports these properties. However, authors also conducted a literature survey to provide the supporting information about the quercetin carcinogenicity, which is depicted as follows.

Quercetin is a naturally occurring flavonol that has a long history of use in the human diet. It can be extracted from plants by isolating the quercetin glycosides, then hydrolysing them to produce aglycone, and then purifying it. Several pharmacological properties like antioxidant, anticancer, antiviral, antidiabetic, anti-inflammatory, antiprotozoal, antiarthritic, cardioprotective, anti-Alzheimer's, chelation, bacteriostatic, anti-carcinogenic, and wound-healing properties have been reported with the quercetin **[2-4]**.

However, recent developments prove that consumption of quercetin has been a debatable concern. The International Agency for Research on Cancer (IARC) stated in 1999 that quercetin should not be classified as a human carcinogenic agent **[5]**. However, *in vitro* studies suggest quercetin can have a minor detrimental impact on foetal growth **[6]**. In the Ames test, reports of mutagenicity in the 1970s raised worries about its safety. In support of this, several studies have been reported with *in vitro* and *in vivo* mutagenicity **[7]**. Quercetin has also been reported to cause a slight increase in the incidence of malignant tumours in the early offspring of mice lacking DNA repair in *in vivo* trials **[8]**. In human clinical trials, quercetin was safe and well-tolerated. Quercetin administration at a dose of >1000 mg/day for several months had no adverse effects on serum electrolytes, renal, haematological, and liver function blood parameters **[9]**.

The majority of *in vivo* studies have concluded that quercetin is non-carcinogenic and may even protect against Geno toxicants. When ingested quercetin was subjected to first-pass metabolism in the intestine and liver, it was nearly entirely metabolised, lowering the risk of carcinogenicity and toxicity. There has been no evidence of toxicity at oral supplemental doses >1000 mg per day for up to three months; however, research on long-term safety at large doses is missing **[8]**.

References

1. Morris, K.F.; Billiot, E.J.; Billiot, F.H.; Gladis, A.A.; Lipkowitz, K.B.; Southerland, W.M.; Fang, Y. A molecular dynamics simulation study of the association of 1, 1′-binaphthyl-2, 2′-diyl hydrogenphosphate enantiomers with a chiral molecular micelle. *Chem. Phys.* **2014**, *439*, 36-43.

2. Batiha, G.E.; Beshbishy, A.M.; Ikram, M.; Mulla, Z.S.; El-Hack, M.E.; Taha, A.E.; Algammal, A.M.; Elewa, Y.H. The pharmacological activity, biochemical properties, and pharmacokinetics of the major natural polyphenolic flavonoid: quercetin. *Foods*. **2020**, 374.

3. Salehi, B.; Machin, L.; Monzote, L.; Sharifi-Rad, J.; Ezzat, SM.; Salem, M.A.; Merghany, R.M.; El Mahdy, N.M.; Kılıç, C.S.; Sytar, O.; Sharifi-Rad, M. Therapeutic potential of quercetin: new insights and perspectives for human health. *Acs Omega*. **2020**, 5, 11849-118472.

4. Maalik, A.; Khan, F.A.; Mumtaz, A.; Mehmood, A.; Azhar, S.; Atif, M.; Karim, S.; Altaf, Y.; Tariq, I. Pharmacological applications of quercetin and its derivatives: a short review. *Trop. J. Pharm. Res.* **2014**, *13*, 1561-1566.

5. Utesch, D.; Feige, K.; Dasenbrock, J.; Broschard, T.H.; Harwood, M.; Danielewska-Nikiel, B.; Lines, T.C. Evaluation of the potential in vivo genotoxicity of quercetin. *Mutat. Res. Genet. Toxicol. Environ. Mutagen*. **2008**, *654*, 38-44.

6. Pérez-Pastén, R.; Martínez-Galero, E.; Chamorro-Cevallos, G. Quercetin and naringenin reduce abnormal development of mouse embryos produced by hydroxyurea. *J. Pharm. Pharmacol.* **2010**, *62*, 1003-1009.

7. Vanhees, K.; de Bock, L.; Godschalk, R.W.; van Schooten, F.J.; van Waalwijk.; van Doorn-Khosrovani, S.B. Prenatal exposure to flavonoids: implication for cancer risk. *Toxic. Sci.* **2011**, *120*, 59-67.

8. Harwood, M.; Danielewska-Nikiel, B.; Borzelleca, J.F.; Flamm, GW, Williams GM, Lines TC. A critical review of the data related to the safety of quercetin and lack of evidence of in vivo toxicity, including lack of genotoxic/carcinogenic properties. *Food Chem. Toxicol*. **2007**, *45*, 2179-205.

9. Wang, Y.H.; Chao, P.D.; Hsiu, S.L.; Wen, K.C.; Hou, Y.C. Lethal quercetin-digoxin interaction in pigs. *Life. Sci.* **2004**, *74*, 1191-1197.
